# Multimorbidity-associated emergency hospital admissions: a “screen and link” strategy to improve outcomes for high-risk patients in sub-Saharan Africa: a prospective multicentre cohort study protocol

**DOI:** 10.3310/nihropenres.13512.1

**Published:** 2024-01-18

**Authors:** Stephen A. Spencer, Alice Rutta, Gimbo Hyuha, Gift Treighcy Banda, Augustine Choko, Paul Dark, Julian T. Hertz, Blandina T. Mmbaga, Juma Mfinanga, Rhona Mijumbi, Adamson Muula, Mulinda Nyirenda, Laura Rosu, Matthew Rubach, Sangwani Salimu, Francis Sakita, Charity Salima, Hendry Sawe, Ibrahim Simiyu, Miriam Taegtmeyer, Sarah Urasa, Sarah White, Nateiya M. Yongolo, Jamie Rylance, Ben Morton, Eve Worrall, Felix Limbani, MultiLink Consortium

**Affiliations:** 1 Malawi-Liverpool-Wellcome Programme, Blantyre, Malawi; 2 Liverpool School of Tropical Medicine, University of Liverpool, Liverpool, England, UK; 3 Kilimanjaro Clinical Research Institute, Moshi, Tanzania; 4 Muhimbili University of Health and Allied Sciences, Dar es Salaam, Tanzania; 5 Humanitarian and Conflict Response Institute, The University of Manchester, Manchester, England, UK; 6 Duke University School of Medicine, Duke University, Durham, North Carolina, USA; 7 The Kamuzu University of Health Sciences, Blantyre, Malawi; 8 Kilimanjaro Christian Medical University College, Moshi, Tanzania; 9 Achikondi Women Community Clinic, Lilongwe, Malawi

**Keywords:** Multimorbidity, non-communicable diseases, hospital care, sub-Saharan Africa, health related quality of life, patient costs, health system costs

## Abstract

**Background:**

The prevalence of multimorbidity (the presence of two or more chronic health conditions) is rapidly increasing in sub–Saharan Africa. Hospital care pathways that focus on single presenting complaints do not address this pressing problem. This has the potential to precipitate frequent hospital readmissions, increase health system and out-of-pocket expenses, and may lead to premature disability and death. We aim to present a description of inpatient multimorbidity in a multicentre prospective cohort study in Malawi and Tanzania.

**Primary objectives:**

Clinical: Determine prevalence of multimorbid disease among adult medical admissions and measure patient outcomes. Health Economic: Measure economic costs incurred and changes in health-related quality of life (HRQoL) at 90 days post-admission. Situation analysis: Qualitatively describe pathways of patients with multimorbidity through the health system.

**Secondary objectives:**

Clinical: Determine hospital readmission free survival and markers of disease control 90 days after admission. Health Economic: Present economic costs from patient and health system perspective, sub-analyse costs and HRQoL according to presence of different diseases. Situation analysis: Understand health literacy related to their own diseases and experience of care for patients with multimorbidity and their caregivers.

**Methods:**

This is a prospective longitudinal cohort study of adult (≥18 years) acute medical hospital admissions with nested health economic and situation analysis in four hospitals: 1) Queen Elizabeth Central Hospital, Blantyre, Malawi; 2) Chiradzulu District Hospital, Malawi; 3) Hai District Hospital, Boma Ng’ombe, Tanzania; 4) Muhimbili National Hospital, Dar-es-Salaam, Tanzania. Follow-up duration will be 90 days from hospital admission. We will use consecutive recruitment within 24 hours of emergency presentation and stratified recruitment across four sites. We will use point-of-care tests to refine estimates of disease pathology. We will conduct qualitative interviews with patients, caregivers, healthcare providers and policymakers; focus group discussions with patients and caregivers, and observations of hospital care pathways.

## Plain Language Summary

**Background:**

In sub-Saharan Africa, multimorbidity (defined as people living with two or more chronic health conditions) is increasing due to high infectious ( *e.g.*, human immunodeficiency virus (HIV)) and non-communicable ( *e.g.*, high blood pressure and diabetes) disease burdens. Multimorbidity increases as people live longer and can be worsened by HIV and HIV-medications. Patients delay seeking help until they are severely ill, meaning hospitals are key to healthcare delivery for chronic diseases, however hospital clinicians often focus on a single disease. Failure to identify and treat multimorbidity may lead to frequent readmissions, high costs, preventable disability and death.

**Aim:**

This cohort study is the first in a three-phase study with the overarching goal to design and test a system to identify patients suffering from multimorbidity when they seek emergency care in sub-Saharan African hospitals. This could improve early disease treatment (reducing death), ensure better follow-up and prevent disability, readmission and excess costs. The cohort study aims to determine multimorbidity prevalence, outcomes and costs. The results will help us co-create with key stakeholders the most cost-effective way to deliver improved care for patients before testing this strategy in a randomised trial.

**Methods in Brief:**

In Malawi and Tanzania, we will identify multimorbidity among patients admitted to hospital (focusing on high blood pressure, diabetes, HIV and chronic kidney disease), by enhancing diagnostic tests in hospital departments treating acutely admitted medical patients. With the help of healthcare professional, patients and community groups we will find how best to link patients to long-term care and improve self-management. After mapping health system pathways, we will work with stakeholders (policymakers, healthcare worker representatives, community and patient groups) to co-develop an intervention to improve outcomes for patients with multimorbidity. This study will allow us to collect clinical, health economic and health system data to inform this process.

## Introduction

### Background and rationale

Non-communicable diseases (NCDs), which can be accelerated by human immunodeficiency virus (HIV) infection and its therapy, are a major health problem in sub-Saharan Africa (sSA) ^1^. Diagnosis is frequently delayed until emergency hospital presentation, when people are already severely unwell ^2^. In hospitals, unrecognised multimorbidity (“the co-existence of two or more chronic conditions” ^3^ results in frequent re-admission, high out-of-pocket expenses, disability and preventable death ^4^. In sSA, co-existent infectious and non-communicable disease pathology is common ^5^. Some studies suggest a higher burden of multimorbidity among females compared to males ^6, 7^. Existing risk stratification and screening frameworks from High Income Countries are poorly calibrated for people in sSA and underestimate severity. For example, diabetes and hypertension are more prevalent in African populations at younger ages ^8^. Acute medical admission is frequently the index presentation of multimorbid disease in areas of poverty ^9^.

The patient pathway in sSA regions may be protracted and complex ^10^. People frequently seek healthcare late for reasons including opportunity cost, poor health literacy and prior consultation with informal care providers. This leads to high prevalence of poorly controlled multi-morbid disease in hospital emergency care where recognition of multimorbidity is delayed or prevented by weak triage systems, resource limitations, and a focus on a single primary diagnosis ^11^. Commitments to Universal Health Coverage and the World Health Organization Model List of Essential Medicines ^12^ mean that HIV and NCDs are treatable through improved access to essential medicines. However, significant barriers include a lack of patient empowerment and health literacy, shortage of healthcare workers (HCW) and limited training ^13^.

Malawi (low income) and Tanzania (low-middle income) are neighbouring countries in the sub-Saharan African region that have experienced dramatic increases in life expectancy between 2000 and 2020 (Malawi 45.6 to 64.3 and Tanzania 50.8 to 65.5 years of age) ^14, 15^. This has been accompanied by increased rates of non-communicable diseases such as hypertension and type 2 diabetes, however, the health systems of both countries are currently weighted toward communicable disease control ^16^ with insufficient provision for the management of multimorbid conditions. Roll-out of anti-retroviral treatment for HIV has been a success, and as patients with HIV live longer, there is increasing risk of frequent and complex interaction between HIV and NCDs; in particular hypertension and diabetes ^17^.

Based on data and clinical reports of medical patient cohorts in Tanzania and Malawi, three frequently co-existing diseases warrant immediate focus: HIV infection, hypertension and diabetes ^18– 23^. A systematic review and meta-analysis also confirmed high prevalence of these conditions among medical in-patients in sub-Saharan hospitals (HIV: 36.4%; hypertension: 24.7%; diabetes: 11.9%) ^24^, however, these data are limited in scope and taken from multiple sources, targeted to single rather than multimorbid diagnoses. Chronic kidney disease was another common condition (7.7% prevalence) ^24^, but which lacked accurate data on chronicity. Inconsistent use of diagnostic methodology and criteria are common in sSA due to under-resourced diagnostic and laboratory services ^24, 25^.

### Objectives

The primary objectives of this mixed methods (clinical, health economics and qualitative) study are:

*Clinical:* Determine the prevalence of multimorbid disease in adults admitted to hospital with acute medical conditions in Malawi and Tanzania.

*Health Economic:* Measure economic costs incurred and changes in health-related quality of life between admission and 90 days post admission.

*Situation analysis:* Describe current care for multimorbidity, enabling and hindering factors to the delivery of patient centred care.

We aim to use the findings to inform the development and design of a randomised controlled trial.

The secondary objectives are:

*Clinical:*

1. Measure prevalence of important pre-selected individual conditions, including chronic non-communicable (hypertension, diabetes mellitus and chronic kidney disease) and communicable (HIV infection) conditions.
2. Measure hospital readmission rate 30 and 90 days after admission.
3. Measure survival 30 and 90 days after admission.
4. Measure a composite of readmission free survival 30 and 90 days after admission.
5. Measure markers of disease control 90 days after admission.
6. Measure the rate of end-organ damage (such as cerebrovascular accident and myocardial infarction).

*Health Economic:*

1. Disaggregate economic costs from patient and health system perspectives and sub-analyse according to the presence of multimorbidity and other socio-economic factors.
2. Model how patients with different multimorbidity incur costs as they pass through the health system.
3. Analyse changes in Health-related Quality of Life according to presence of different diseases.
4. Estimate the potential cost-effectiveness of a selected intervention to improve diagnosis and treatment for people with multimorbidity.

*Situation Analysis:*

1. Describe healthcare pathways for adults during and after acute medical admission using structured observational data during patient admission.
2. Qualitatively describe (i) patients’ level of health literacy related to their own conditions and (ii) healthcare engagement for patients with multimorbidity using key-informant interviews.

### Overarching study hypothesis

For adults admitted to hospital in Malawi and Tanzania, co-existing medical conditions occur at a rate where screening is worthwhile at the individual and health system level (predefined at ≥5% prevalence).

## Protocol

### Study phases and design

**Figure 1.**
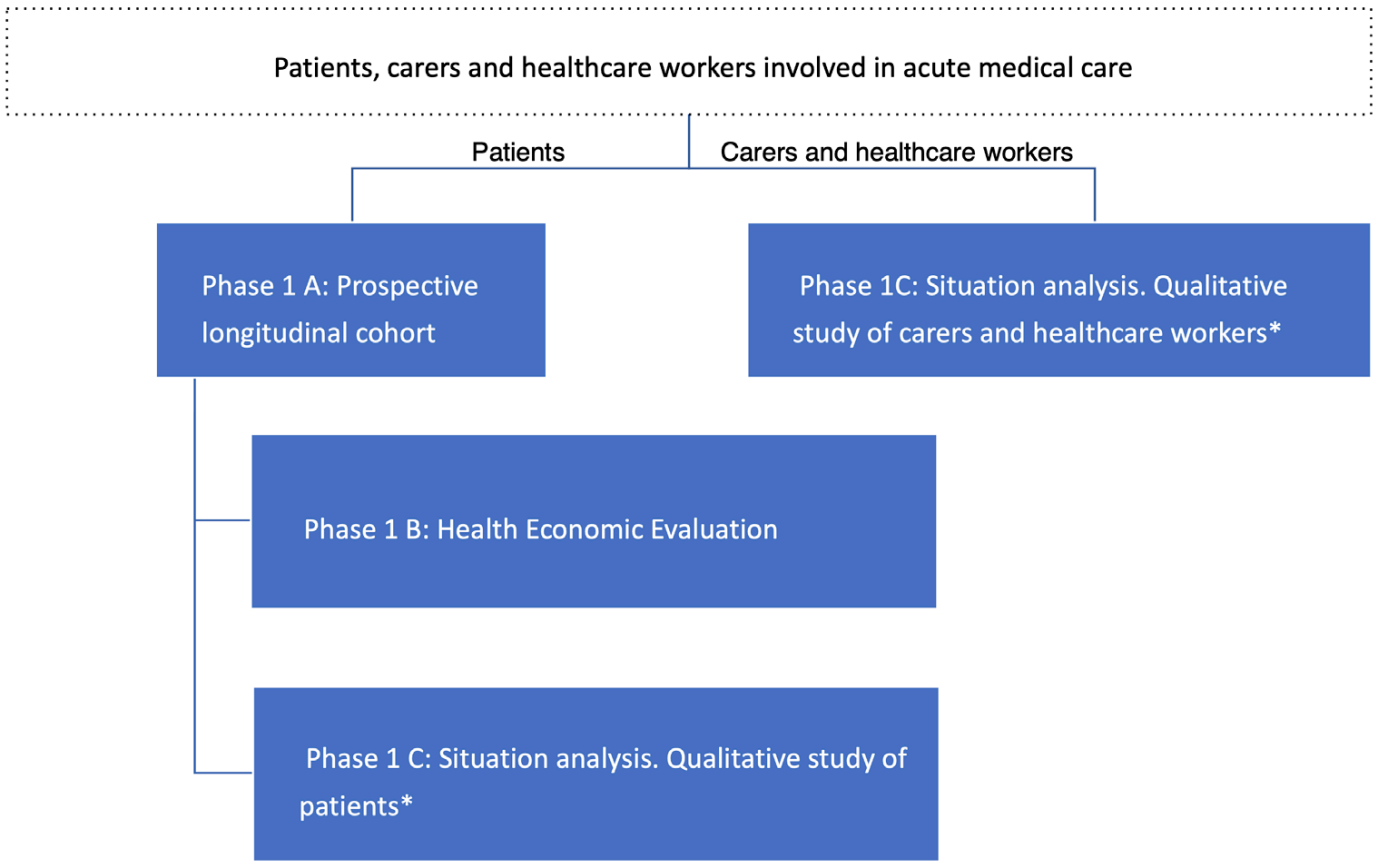
Study layout. * qualitative study requires separate consent.

Our funded programme of work is divided into three phases: (1) a prospective longitudinal clinical cohort study of acute medical admissions with nested health economics study and situational analysis, (2) co-creation of a complex intervention aiming at delivering improved care for people with multimorbidity and (3) a cluster randomised trial with nested process and economic evaluation to evaluate the intervention. This protocol focuses on phases 1 and 2. Phase 1 incorporates three distinct methodologies (see Figure 1): 1 A) Prospective longitudinal cohort study; 1 B) health economic evaluation focussing on costs (patient and health-system) and consequences (health related quality of life); 1 C) patient-centred situation analysis with mixed qualitative methods including key informant interviews, focus group discussions and observation of patient care pathways. Phase 2 incorporates intervention cost-effectiveness modelling, to support co-creation, design and selection of an intervention to be evaluated subsequently through a cluster randomised clinical trial (phase 3).

### Setting

This study will be conducted in four sites across two countries (Malawi and Tanzania). Within each country we will recruit patients from a central referral hospital and a district hospital to develop generalisable results, broadly applicable within both contexts. The study sites are: Queen Elizabeth Central Hospital, Blantyre, Malawi; 2) Chiradzulu District Hospital, Southern Region, Malawi; 3) Hai District Hospital, Tanzania; 4) Muhimbili National Hospital, Dar-es-Salaam, Tanzania. The study is planned to take approximately 15 months (12 months recruitment, with an additional three months to complete follow up) and will be conducted in parallel in all sites.

### Patient and Public Involvement

We partnered with established organisations working in the sphere of NCDs in Malawi and Tanzania. These include the Malawi and Tanzania NCD Alliances, Community for Disease Prevention and Management, Malawi Health Equity Network and community research advisory boards. These structures have direct contact with beneficiaries and community leaders. Prior to the development of the study, we conducted community forums with patients with NCDs, care givers, leaders of peer support groups and members of community research advisory board. These groups reflected on their experience with multimorbidity in their home, barriers and facilitators to diagnosis, treatment, and care for multimorbidity in health facilities and proposals for improvement. Their experiences informed the development of our situation analysis questions. We also engaged policy stakeholders from Ministries of Health in both countries in a need analysis process. This helped us to gain: the core national indicators for monitoring progress of NCDs and the status of their ongoing progress; national, local and community setup of structures for governing and managing NCD and multi-morbidity services: current NCD policies, programmes, and implementation strategies. Proposals and issues that came up from both the policy and community consultation will be part useful during the second and third stage of the study when we develop and implement the intervention (with continued engagement). The needs analysis helped in identifying key stakeholders whom to engage during this period as community and policy members from community advisory boards and national advisory groups.

### Participants

***Phase 1 A: Prospective longitudinal cohort study*.** Patients will be screened for enrolment at the primary point of entry for hospital admission. Cohort study participants will be consecutively recruited within 24 hours of emergency presentation, and stratified recruitment across four sites. Eligibility criteria are as follows:

Inclusion:

1. Adult patients (aged ≥18 years).
2. Decision to admit to hospital.
3. Physician diagnosis of an acute medical disorder.
4. Residence within the study catchment area (predefined for each hospital site).
5. Contactable on discharge by phone (either directly or through a carer).

Exclusion:

1. Pregnancy (other research groups are specifically addressing this).
2. Planned medical admission.
3. Detainees or prisoners.
4. Admission for primary trauma, obstetric or gynaecological condition.
5. Patient or carer declines consent to take part.

The study procedures and schedule for follow-up are described in Table 1.

**Table 1.** Study procedures and sampling schedule.

Study Visit	A	B	C	D	E	F	G
Day post admission	0	2	5	7	Discharge	30	90
Deferred consent	x						
Consent (verbal)	x	x	x	x	x		x
Consent (written)	3	3	3				
Vital signs (including blood pressure)	x	x	x	x	x		x
Medical history, function (including grip strength) and outcome assessment	x					4	x
Screen for Adverse Events (AEs)	x	x	x	x	x		x
HIV POC *	x						x
Where HIV, VL, urinary LAM, serum CrAg *	x						1
Blood glucose POC	x	x	x	x			x
HBA1c POC	x						x
Creatinine POC	x						x
Urinary dipstick	x						x
Serum save 5mL)	x						x
Situation analysis	x	x	x	x	x		x
Patient and health system costs					x	x	x
EQ5D questionnaire	x					x	x
Qualitative interviews					x		x

AE: adverse event; POC: point of care blood test; HIV: human immunodeficiency virus; VL: viral load; urine LAM: urinary lipoarabinomannan; CrAg: cryptococcal antigen; HbA1c: haemoglobin A1c (glycated haemoglobin). Additional contact with the participants may be made up to approximately 12 months after hospital admission to check the longer-term outcomes. This will be by telephone where possible. Visit F (hospital discharge) visit data will be collected, this may occur at any point before or after the 2, 5 and 7 day follow up visits. Visit G (90 day) will occur in a specific research outpatient follow-up clinic. There will be flexibility of ±10 working days for this follow up visit to take place. * Diagnostic HIV test and/or HIV viral load will be collected as part of routine clinical service. Parentheses indicate only if required to confirm diagnosis (rely on concurrent or earlier test). 1: Samples for urinary LAM and serum CrAg test will be collected and processed as part of routine clinical service to screen for opportunistic infection in line with WHO guidance at admission. 2: Qualitative interviews will take place with healthcare workers in a portion of treating providers after patient discharge. Qualitative interviews will take place with a proportion of patients after follow up visit at 90 days. 3: Written consent will be taken at the earliest possible opportunity, either directly from the patient or from a proxy (if patient lacks capacity). 4: Telephone or home visit follow-up (in order to maximise retention and data completeness)

***Phase 1 B: Health economic evaluation*.** One third of participants recruited to the cohort study will be randomised to the health economics study, with follow-up and data collection as per cohort study, or as detailed in section (Variables, below).

***Phase 1 C: Situation analysis*.** IDIs with patients and caregivers will include patients purposively sampled from the overall cohort whilst still an in-patient, at the point of hospital discharge or during the 90-day follow up clinic. Carers will include those supporting patients whilst they are being treated in hospital or who supported a patient who died (during their hospital stay or within 90 days after admission). During interviews, caregivers will be linked/matched to patients in approximately half of the cases (caregivers of the patients we will interview). This will help to diversify the data and facilitate recall for patients who may have experienced reduced capacity during the early stages of hospital admission.

FGDs will be conducted with patients who have recovered from acute medical admission and their caregivers, in the community, after hospital discharge. We will hold female and male specific FGDs to ensure equal participation, especially for women who may be inhibited from disclosure in the presence of men. We will approach and recruit the caregivers of acute medical patients (those staying full time with the patients at the hospital and providing day to day care) during their hospital admission. Patients and caregivers will take part in either FGD or in-depth interviews, but not both.

IDIs with healthcare workers and policy makers will include HCWs directly involved with acute medical patients – physicians (senior clinicians (consultants, juniors), nurses, ward attendants, reception clerks and/or frontline administrative staff. Policy makers will include those working in decision-making roles at the district and national hospitals and service delivery level of the government-funded health system.

Inclusion criteria

1. Patients (≥18 years) recruited from cohort study (separate consent sought for this sub-study).
2. Carers/guardians caring for patients within the cohort study.
3. Healthcare workers who provide direct care for patients recruited to the cohort study.
4. Healthcare workers taking part in the training as part of the programme roll-out.
5. Policymakers that have occupied their current or previous policymaker position for 3 months or more.

Exclusion criteria

1. Patients and carers who do not speak a language understood by one of the field research team (not anticipated, as team speak both English and the predominant local languages).
2. Patients, carers and healthcare workers who are judged likely to be negatively affected by participation *e.g.*, due to distress.
3. Patients who are too sick to be out of bed and participate in an interview in a private area.
4. Policymakers that are yet to be involved in policy development.

### Consent process

During screening, participants will give informed written consent to their data being used for clinical cohort and HE study (consented together prior to allocation to HE study) and the modelling (Phase 2). In cases where prospective consent not possible, deferred consent model will be offered (to allow those who initially lack capacity to be represented in the cohort. A separate written consent will be sought for the participants recruited to the situation analysis including health care workers.

We will obtain informed consent from patients if their physical and mental capacity allows based on the following questions: 1) can the patient retain information; 2) can the patient weigh the information; 3) can the patient communicate a decision. Where there is uncertainty, these can be assessed through: 1) asking the patient to reflect the given information back to the study staff, including the purpose, procedures and risks of the study; 2) asking the patient to convey the alternatives of participation in the study, including understanding the voluntary nature of inclusion.

For fully competent patients, an information sheet will be provided, the study will be discussed and written consent obtained. Illiterate patients will be read the information sheet and mark the consent form with either a cross or thumbprint to indicate their agreement in the presence of an independent witness. If a patient is unable to give consent, we will obtain consent from a proxy (a relative or representative) in the same manner. Where the patient subsequently regains capacity, we will approach them to discuss and obtain retrospective consent. If no proxy is available and local regulations allow, we will use a deferred consent process. If deferred consent is used, we will tell the patient about the study as soon as possible and obtained consent for use of the data collected. If the patient does not have capacity or if a significant delay to regaining capacity (>7 days) is anticipated, then we will seek retrospective assent from their proxy.

Justification for this consent model is that patients stand to benefit from early use of enhanced diagnostics using CE marked and commercially available point of care tests. Delayed diagnosis in this context has potentially detrimental effects for patients who otherwise would not have access to the enhanced diagnostic package. Patients will be at minimal risk through study procedures. From a scientific perspective, this model will reduce the risk of recruitment bias. There is precedence for deferred consent in the Malawian context with existing data describing the acceptability of this approach ^26^. Deferred consent has also been used in Tanzania for a randomised controlled trial of tranexamic acid *versus* placebo after post-partum haemorrhage ^27^. All participant information sheets and consent forms will be translated into local languages (kiSwahili in Tanzania, and Chichewa in Malawi).

### Variables

***Phase 1 A: Prospective longitudinal cohort study*.** The main outcome is to define the characteristics of multimorbidity. We will employ enhanced point of care test (POC) diagnostics for patients admitted to the study to determine the prevalence of multimorbid diseases of interest (specifically HIV, hypertension, diabetes and chronic kidney disease). All POCs used in the study are CE marked and commercially available. We will make the results of these tests available to treating healthcare providers using a standardised proforma. No treatment recommendation will be provided with this information and clinicians will be able to interpret and use this information independently. Structured data extraction of clinical information (including potential predictors of patient outcomes) will be performed at intervals (described in Table 1 from patient records.

***Phase 1 B: Health economic evaluation*.** To explore the impact of socioeconomic status (SES) on patient costs, clinical outcomes and health-related quality of life (HRQoL) we will calculate wealth scores and use these to assign all clinical cohort study participants to SES quintiles. For participants randomised to the health economic study, we will collect data on patient costs at admission (in person), discharge (in person), 30 and 90 days post discharge (in person or by telephone). Health system costs for patients in the cohort study will be modelled using top down and bottom up costing methods. HRQoL will be estimated in quality adjusted life years (QALYs).

***Phase 1 C: Situation analysis*** In depth interviews (IDI) and focus group discussions (FGD) with patients recruited to the cohort study, and their care givers will be conducted to understand their experiences, preferences, and priorities, including level of health literacy and engagement with healthcare providers for long term management of chronic conditions. Exploration of detailed experiences from patients and carers will provide specific concrete examples of their expectations, and any concerns they have, as well as their priorities and perceptions of how the hospital currently manages acute medical patients with multimorbidity, problems and how best to address them.

In-depth interviews and structured discussions with health care providers who provided direct care for patients recruited in the cohort study will be conducted to determine “nodes” in the treatment pathway where an intervention could effectively be delivered. Key informant interviews with policymakers at national and district level will explore the pathways of health care for people living with multimorbidity, their perspectives of the policy environment around the management of multimorbidity, and how an intervention could be incorporated into current practice.

Observations of patient care pathways in a purposefully selected sample of participants, will describe the patient pathway, and the effect of hospital context on acute medical care for multimorbid disease. We will observe the functioning of the wards, the relationship among healthcare workers (HCWs) and between HCWs and the patients. We will conduct semi-structured observation of hospital activities, patient pathways and patient consultations for adults admitted with acute medical conditions.

### Data sources and measurement

***Phase 1 A: Prospective longitudinal cohort study - baseline and clinical data*.** The primary outcome of the clinical cohort study will be to collect data on conditions known to be highly prevalent among medical in-patients in sSA, and are likely to be disease constituents of multimorbidity. Following results from a recent systematic review ^24^, we will therefore focus on HIV, hypertension, diabetes and chronic kidney disease. Importantly these conditions can be diagnosed using available point-of-care tools. Although heart failure is also a common in these settings, diagnosis is technically more challenging and is being explored separately. We will apply standardised point of care testing to participants and record these primary outcome conditions both in the electronic data capture tools and in the patient notes, making these explicitly available to treating clinical teams.

HIV: HIV will be tested using locally available HIV rapid diagnostics tests. For HIV positive patients we will also test the HIV viral load using existing laboratory infrastructure in each site.

Hypertension: In patients with a regular pulse rhythm, automated electronic sphygmomanometer will be used. In patients with an irregular pulse rhythm, manual aneroid sphygmomanometer will be used. Patients will be semi-recumbent or supine, legs uncrossed, relaxed for at least five minutes after the start of the consultation, and not talking. On the first visit, the blood pressure will be taken from both arms and using the arm with the highest reading thereafter. At each visit two readings will be taken from this arm, recording the second reading.

Diabetes: HbA1c levels will be measured using the POC Hemocue ^®^ HbA1c 501 Analyzer and cartridges (HemoCue AB, Ängelholm, Sweden).

CKD: Abbott iSTAT point-of-care device (Abbott Point of Care Inc, Illinois, USA) with Chem8+ cartridges will be used to measure creatinine from venous blood, directly following the manufacturer’s iSTAT User Guide.

Other conditions are expected to contribute to multimorbidity, for example hearing and visual impairment, causes of impaired mobility such as injury at birth, or disability from trauma, surgical procedures and others later in life. A key criterion for inclusion in the planned intervention study will be potential for improved morbidity or mortality from linkage to care. We will therefore collect data on disability using standardised questionnaires, with key components from Washington Group on functioning, Patient Health Questionnaire (PHQ)-9 for depression in addition to data tools used in previous cohort studies for the collection of standardised clinical information, and allow this to inform the intervention design.

Clinical outcomes, coded from a structured case summary and the available medical notes, will be established as follows:

1. Medical diagnosis and supporting evidence.
2. In-patient treatment of these diagnoses, including time of appropriate treatment establishment.
3. Vital signs, including pulse oximetry (using a A310 finger oximeter), respiratory rate, pulse rate, blood pressure and temperature.
4. Routine laboratory data supporting these diagnoses (such as urine lipoarabinomannan [LAM], serum CrAg).
5. Clinical outcomes will also be ascertained at hospital discharge, 30 days and 90 days as:
6. Death (coded from all available records).
7. Readmission to hospital.

Data will be collected by a study nurse using electronic data capture. This will describe the patient characteristics, medical history and ‘patient journey’ through the healthcare system. Age, date of birth and sex will be documented from patient reports and/or clinical notes. To ensure complete data collection, we will prospectively collect data on vital signs, details of resuscitation, medication administration, results of clinical, laboratory, and radiologic examinations. All clinically relevant findings will be entered into the medical notes to ensure availability to the responsible clinicians.

As a component of the functional assessment, we will measure hand grip strength at day 90 follow-up using a GRIPX Digital Hand Dynamometer, which is a known predictor of health and overall strength ^28^. Grip strength will be measured seated on a chair without elbow rests, with the elbow loosely flexed at 90 ^0^, and the wrist in a neutral position ^29, 30^. Grip strength will be measured as the mean of three tests on both hands with 60 seconds of recovery between each attempt.

***Phase 1 B: Health economic data sources and measurement*.** All consenting study participants will be asked asset ownership, house construction and demographic questions extracted from the most recent relevant national Demographic and Health Survey (DHS). Patient cost data will be captured before, during, and for 90 days after hospital admission using an amended version of the STOP-TB tool, a widely-used questionnaire for measuring TB patient costs ^31^. We will capture direct medical costs ( *e.g.*, drugs, diagnostics, consultation and inpatient fees), non-medical costs ( *e.g.*, food and transport), indirect costs (lost time for patients and guardians). Where a patient is readmitted to hospital, we will also capture these costs. Costs will be captured in local currency and presented in 2024 United States dollars.

Health system costs will be collected using top-down and bottom-up (micro) costing methods. We will develop and validate (with relevant hospital staff) a visual model of individual patient pathways through the study hospitals. This will be supplemented with data on waiting times, and time spent with various health workers from the sample of patients that are included in observations conducted as part of the situation analysis. Costs for each stage of the patient pathway (triage, admission, diagnostics, treatment, discharge, readmission *etc*.) will be based on unit costs for tests, staff time, hospital bed days and other inputs, obtained from hospital data, tests and consumables suppliers, and Ministry of Health (MoH). Any other costs deemed relevant during the data collection stage will also be included. Top-down costing data on hospital overheads (utilities, building costs, maintenance, *etc*.) will be obtained for each hospital.

HRQoL data will be collected using the EQ-5D-5L questionnaire in English, Chichewa (Malawi) and kiSwahili (Tanzania). The questionnaire will be administered, at admission, discharge, 30 and 90 days post admission in person or (day 30) by phone (as for patient costs).

***Phase 1 C: Situation analysis*.** Experienced social science fieldworkers fluent in the predominant local language (kiSwahili in Tanzania and Chichewa in Malawi) will collect data with guidance from the clinical academic teams. Observation tools and interview topic guides will be piloted to ensure understandability, and promote effective dialogue and data generation. We will conduct one pilot observation and interview for each of the patient, caregivers and HCW groups. After this, early observation notes and interview transcriptions will be reviewed by the senior academic team to verify and refine as required the phrasing of questions and level of probing. Interviews and focus group discussions will be audio-recorded. During focus group discussions, a note-taker will assist the facilitator and record differences and similarities among group members in their reaction and response to questions. These steps will promote diversity in data collection.

### Sample size

***Phase 1 A: Prospective longitudinal cohort study*.** The primary outcome will be disease prevalence. At 5% prevalence, a precision of 1.5% and power of 90% at α=0.05 is expected (precision will reduce as actual estimate rises towards 50%). We will use 5% prevalence as the lower boundary for inclusion of the disease during intervention development. We expect that HIV, hypertension and diabetes will meet this threshold. The cohort will allow us confidence to decide on the inclusion of chronic kidney disease. Prevalence will be assessed in both countries at the district and central hospital level to determine if findings are shared across settings. Given these assumptions, sample size is 1544 patients, with a target of 1600 to account for drop out. We will recruit these patients across the four hospitals (with numbers varying between sites due to different patient loads).

***Phase 1 B: Health economic evaluation*.** A randomised sub-sample of one third of the cohort study participants will be selected for participation in patient costing and HRQoL analysis (approximately 133 participants per hospital).

***Phase 1 C: Situation analysis*.** Sampling strategies for patients and caregivers is designed to include those individuals representing: those admitted for the first time and those who are returnees; those on HDU, ICU and ordinary wards; different age categories; male and female. We will ensure inclusion of patients with multiple known pathologies representing “multimorbidity” according to the final definition. Sample sizes for interviews are preliminary; they will continue until a point of saturation is reached. Table 2 shows estimates of the number of patient and carer interviews required. For healthcare workers, we will include different cadres working in different departments.

**Table 2.** Number of in-depth interviews, focus group discussions and structured discussions.

PER HOSPITAL SITE	In-depth interviews	Focussed group discussions	Structured discussions
Patients with multimorbidity *	10	24	10
Caregivers to patients with multimorbidity *	10	24	10
Doctors	2	-	4
Nurses	4	-	8
Ward attendants	2	-	6
Reception / frontline administration staff	2	-	2
Total	30	48	40

* Patients and caregivers will not be the same group during IDIs and FGDs. IDI, In depth interviews; FGD, focus group discussion.

Observations will be conducted at different days of the week and at different times of the day including both the morning and afternoon hours (examining variation over time). In each day, the observer will spend four to five hours conducting these hospital observations with a further two to three hours of reflection to document and expand the observation notes. We will observe 40 patients consultations (10 patients per hospital), including a sequence of observations throughout their follow-up to establish pathways, timings and bottlenecks in the system. Observers will balance male and female patients, observing different consultation rooms and patients admitted at different times of day.

### Analysis

***Phase 1 A: Prospective longitudinal cohort study - quantitative statistical analysis*.** We will report the study in accordance with STROBE guidelines. A CONSORT diagram will summarise participant enrolment and follow-up. We will report descriptive statistics, including N (sample size of analysis population), n (sample size of analysis population without missing values). For continuous data we will report the mean and standard deviation for normally distributed data, or median and interquartile range for data that are not normally distributed. The proportion of observed levels will be reported for all binary and categorical measures alongside corresponding exact, binomial exact 95% confidence intervals (CIs) for proportions when appropriate. Results will be disaggregated by sex. Where appropriate, we will use multiple imputations using chained equations for missing data.

Will provide in-depth baseline epidemiologic and clinical management data across the different sites. Similarities and differences across sites will be recorded. Analysis will include evaluation of the association between clinical management and patient outcomes. Univariate and multivariate strength of association between variables and patient outcomes will be tested by logistic regression modelling.

Prevalences will be given as a proportion with 95% confidence intervals of the population estimate. Combinations of more than one will be assessed for their “joint prevalence” in order to understand multimorbidity disease clusters.

***Phase 1B: Health economic*.** Principle components analysis (PCA) will be used to construct wealth scores using individuals’ asset and other data, and wealth quintiles in the study population. Additionally, participants will be assigned to quintiles in reference to most recent DHS survey, allowing socioeconomic assessment of the study population relative to the national population.

Patient costs will be estimated for each individual patient including any costs incurred by their guardians in accompanying them to hospital or subsequent health care visits. Indirect costs ( *e.g.*, lost time for paid or unpaid work, including housework) will be estimated based on the number of days of reported inability to work multiplied by the pre-disease wage given by patients during their baseline interview. The patient pathway model will be parameterised with unit costs and used to estimate individual patient and health system costs for a set of the most frequently observed multimorbidity combinations and patient types. Capital costs extending beyond one year will be annualised over their expected lifespan. Results will be presented as means and 95% confidence intervals. Deterministic and probabilistic sensitivity analyses will be conducted to test the robustness of the results. Multiple logistic regression will be used to compare patient costs by multimorbid condition(s) and wealth quintile to identify any significant differences. Additionally, we will examine the relationship between patient costs and age and sex. Change in HRQoL, expressed as QALYs, between admission, day 30 and day 90 interviews will be estimated using the area under the curve method. QALYs will be calculated according to the most relevant available tariff based on both geographical proximity and economic context. In our study we will use Uganda’s tariff for Tanzania, and Zimbabwe’s tariff for Malawi. Updated tariffs will be used if these become available before the analysis. The QALY calculations will also consider mortality during follow-up by attributing the lowest value from each value set (which corresponds to death), from the date of death until the end of the follow-up period. Missing patient cost or HRQoL data will be imputed using multiple chained imputations modelling with a predictive mean matching algorithm and relevant baseline variables.

Health economic analysis will be performed in Stata v15.4 and Excel.

***Phase 1 C: Situation analysis*.** Transcription and translation will be performed in line with local standard procedures. For accuracy, during the first 10 transcriptions a researcher will read the transcribed interviews and focus groups while listening to the audio recordings, and then check translations. Further checks will depend on the quality of these initial evaluations.

For in-depth interviews and focus group discussions, all data collected will be transferred to a qualitative data analysis software package (NVivo), to enable analysis. Data will be analysed following broad deductively defined themes and inductively derived sub-themes. We will employ a combination of thematic and framework coding to compare perspectives between different stakeholders. Two researchers will undertake initial coding of a small number of transcripts, and then discuss and agree themes for further coding. Analysis will be performed concurrently with fieldwork using an iterative approach to identify emerging themes that can be clarified or explored further through later data collection. Data will be triangulated between methods and participant groups to cross-check information and provide a more comprehensive analysis.

### Data management

Consent will be documented on paper or electronic forms. A paper copy will be given to the participant or guardian, and any original paper will be stored in a locked cabinet at the local hospital site, accessible only to the study team. All study data will be collected electronically.

Baseline, follow-up questionnaire data, and physiological observations (including pulse, respiratory rate, blood pressure, and oxygen saturations), and any point-of-care measures will be captured direct to Open Data Kit ^32^ software on devices with password encryption.

Clinical information including presenting complaints, history of presenting complaints, past medical history and drug history will be collected. Data on differential diagnosis and final diagnosis will be collected including clinical actions ( *e.g.*, prescribed medication) and patient response. We will also collect information on prescribed medication at hospital discharge, including where possible out of pocket expenses, health literacy and drug dispensary access (including stock outs).

Laboratory test results will be directly electronically exported from laboratory information systems where possible.

The study will adhere to LSTM (sponsor) data management and security policies, and encryption guidelines. This allows the data from each site to be collated, and presented at summary level in near real-time for the purposes of monitoring recruitment across both countries. During the study, individual participant level data will be available by the study team only, for the purposes of data validation and completeness checking. Access to this will be controlled by the same data security policies, with journaling of data change requests to allow full data audit.

After successful import of external data, and formal closure of the study, data will be fully anonymised by removing identifiers (including embedded electronic data) and granulating data which might enable an individual to be triangulated (area of habitation will be retained at “ward” level, and age rounded to the nearest year).

Data management for the patient costs, and HRQoL will be as per the clinical cohort study. Health system cost data will be collected and stored in custom built Excel spreadsheets.

***Qualitative data*.** Audio files will be stored on a secure network drive and transfer a copy to the project transcription and translation team using encrypted connection. Transcriptions and audio files will only be accessible to the study team. Any paper copies will be stored in a locked filing cabinet. Participant names will not be included in transcripts or file names; instead, participants will be given ID numbers.

Quality assurance measures will include piloting of the approaches described by the topic guides, and informal feedback from all participants on how to improve the structure, style and content of the sessions.

***Data availability*.** ODK will be used to collect data on Android devices. The data from all sites will be synchronised to a server hosted by the Malawi Liverpool Wellcome programme (MLW), Blantyre Malawi. Data will be of high quality and in a format that can be shared with other interested researchers through access of data dictionaries. Handling of data requests will follow the relevant standard operating procedures at MLW. All data will be managed according to a data management plan, and transferred in accordance with a data management plan.

### Ethics

Ethics was obtained from LSTM (21-086; approved on 10.05.2022); College of Medicine Research and Ethics Committee (COMREC), Malawi (P.11/21/3462; approved on 15.10.2021); National Institute for Medical Research (NIMR), Tanzania (NIMR/HQ/R.8a/Vol.IX/4008; approved on 13.05.2022); Kilimanjaro Christian Medical Centre (KCMC) (2570; approved on 15.12.2022).

Our reflexivity statement (available here: doi.org/10.7910/DVN/CKSYSW) describes how we have promoted equity in our international research partnership and authorship within the MultiLink Consortium ^33^.

## Dissemination of findings / results presentation

The findings from this study will be disseminated amongst the scientific community. Results will be feedback and discussed with hospital staff and patient groups. Within the MultiLink consortium, we have dedicated leads for both community and policy engagement, through community advisory boards and policy stakeholder ‘thinktank’ meetings. We intend to publish our findings in peer reviewed scientific journals and present data at appropriate local, national and international conferences. We will produce a close-out report for the local research ethics committees and LSTM (sponsors) at the end of the study and a final report once data are published. In addition, we will produce a lay report of our findings which will be made available to all participants. We will directly summarise the results to all ethics bodies, including COMREC. Data generated from this study will be used to inform design of a complex intervention in collaboration with key stakeholders as a part of structured dissemination activities.

### Study status

This study has started and is currently in the follow-up phase. Statistical code is under development for data analysis, which has not yet been performed.

## Limitations

There are a number of limitations with this study, which we have attempted to mitigate. First, there are a limited number of sites in two countries. In order to generate broadly generalisable results, the chosen sites reflect a mix of tertiary / referral hospitals and district hospitals in each country. Second, we have decided to systematically screen for four conditions (HIV, hypertension, diabetes and CKD) using point-of-care diagnostics. This may miss multimorbidity from other chronic conditions, which require additional infrastructure to confirm diagnoses (such as clinical imaging or enhanced laboratories), but we will collect clinical data on all diagnosed chronic conditions. A recent systematic review indicates a high burden of disease from these four conditions among hospitals patients, which can importantly be diagnosed using available point-of-care tools ^24^. Third, CKD diagnoses require evidence of chronicity of disease at three months and therefore data in this condition is prone to survivorship bias. Fourth, EQ5D-5L data and resulting QALY estimates may not reflect the most accurate picture of HRQoL in our study context. However, through our eCRF, we will collect rich clinical data with additional scales (such as the Patient Health Questionnaire-9 [PHQ-9] screening tool for depression ^34^; and clinical frailty scale ^35^ to explore the possible limitations and interrelationships. Fifth, our planned situation analysis will provide a cross-sectional picture of the health system at the time of the study, which is not able to incorporate the dynamic and adaptive nature of health systems. However, the MultiLink consortium includes researchers from each country and site, with a rich understanding of the context.

## Phase 2 methods: Intervention development

We will share the information and learning from clinical, health economic and qualitative situation analysis and use relevant national treatment guidelines to develop a set of diagnostic and treatment algorithms which seek to optimise care for people with multimorbidity. Group discussions will be held at all recruitment sites to ensure we have accurately understood and synthesized the issues identified through situation analysis, and to explore stakeholder perceptions on the feasibility and desirability of our proposed intervention for the RCT phase.

We will work with stakeholders (frontline healthcare workers, hospital managers and patients/their representatives) in both countries to explain our proposed intervention and get their feedback on practicality, risks and possible improvements. This will consider quantitative resourcing requirements ( *e.g.*, in relation to staffing levels and bed space) as well as feedback from healthcare workers and patients on practical and emotive considerations.

The preferred intervention package will then be tested under Phase 3 (cRCT) under a separate protocol.

## Data Availability

No data are associated with this article.
