# Supplementary material for: Multimorbidity-associated emergency hospital admissions: a “screen and link” strategy to improve outcomes for high-risk patients in sub-Saharan Africa: a prospective multicentre cohort study protocol
Source: NIHR Open Res. 2024 Jan 18;4:2. [Version 1] doi: 10.3310/nihropenres.13512.1 (PMC11320189; doi:10.3310/nihropenres.13512.1)
Supplement: Peer review history

# Reviewer report: Reviewer response for version 1

**DOI:** 10.3310/nihropenres.14663.r31249

**Published:** 2024-04-22

**Authors:** Phuong Bich Tran

**Affiliations:** 1 Nuffield Department of Primary Care Health Sciences, University of Oxford, Oxford, UK

**Summary:**

 The topic of multimorbidity is an important one for study settings in sub-Saharan Africa due to the increasing burden of chronic and infectious diseases, limited healthcare resources, and the need for effective strategies to improve patient outcomes and reduce healthcare costs. This cohort study aims to identify multimorbidity among emergency care patients in sub-Saharan African hospitals, facilitating early treatment, improved follow-up, and reduced disability, readmission, and costs. The study seeks to co-create cost-effective strategies for enhanced patient care.

 While the protocol demonstrates thorough planning and detail, several concerns persist. Foremost is the absence of a theoretical framework guiding the study's conceptualization and implementation, particularly crucial in implementation research. Additionally, the narrow scope of multimorbidity prevalence assessment and the limited focus on a specific study population (i.e., high-risk/severe-stage patients) are notable limitations. The absence of a primary care component, essential for integrated patient-centered care in multimorbidity management, is a critical gap. Despite labelling the project as a "co-creation," there appears to be a perceived limited level of patient and public involvement, warranting further attention and clarification. Please see details below.

**Fairness:**

1. In the protocol, LSTM is designated as the "sponsor". However, the reviewer requests further clarification regarding the involvement, roles, and decision-making power of all partner institutions, as well as the nature of the collaborative efforts. Emphasis is placed on outlining the equitable nature of these partnerships, aligning with the principles of ethical global health research partnerships. Given the predominant role of academic institutions in the Global North as major sponsors of research, and their control over vital resources for global health research (Monette *et al.*, 2021 ^1^), it is imperative to illustrate how power dynamics are addressed to ensure fairness and inclusivity in this project. The reflexivity statement merely states that local researchers are “co-applicants” in the funding application.
2. Patient and public involvement (PPI) is stated to be integrated into the project and the project is described as a process of co-creation. However, the extent to which research can be said to be ‘co-produced’ is a factor of the number of research stages involved in, stakeholder types, the scale of their contribution, and ‘adherence’ to the principles and practice of co-production (see Figure 1 in Beckett *et al.*, 2018 ^2^). It is essential to clarify whether PPI spans all research/project stages of the co-production continuum (e.g., from topic identification to process evaluation) or selective ones, and the level of influence the patients/public have at each stage. Are they considered ‘participants’ or ‘co-researchers’? (If the latter, are any patient/member of the public named author on this publication?) Are they allowed to 'express a view' only or 'contribute to decision making'? (See Levels of Participation Pyramid by Kirby, 1999). If there will be limited involvement of patients and the public, it is important to acknowledge this limitation. Additionally, the term "co-creation" should be used carefully and with nuance, recognizing the extent of involvement and avoiding overstatement of collaborative processes where they may be lacking.

**Theoretical framework**

1. A critical concern is the absence of a theoretical framework to guide the study, underpin concepts and provide a structured foundation for interpreting the findings. This gap becomes particularly pronounced considering the planned development of an implementation strategy aimed at enhancing the quality of care and health outcomes for individuals living with multimorbidity. Relevant to this project may be the Chronic Care Model by Wagner *et al.*, which provides a framework for improving care for patients with chronic conditions. It emphasizes the need for a proactive, planned approach to care that is patient-centered, population-based, and community-oriented.
2. The importance of integrated care in managing multimorbidity, particularly at the primary care level, is well-established. However, this critical aspect is noticeably absent in the protocol, from background/rationale to the development of intervention strategies to deliver improved care and health outcomes for patients with multimorbidity. Primary care is not represented among the study sites and no health workers or patients will be identified from this setting. This omission further underscores the need for a theoretical framework to guide both research and implementation strategies, ensuring comprehensive consideration of all relevant components for effective multimorbidity management.

**Study population:**

1. The term "high-risk patients" is included in the title, is this designation warranted due to the study population being limited to those admitted to emergency care?
2. It remains ambiguous whether patients previously diagnosed with the four preselected conditions at baseline and already undergoing treatment for those conditions will continue to be enrolled in the study. Will they still receive point-of-care (POC) testing? Moreover, is the focus primarily on prevalence or incidence? The emphasis appears to be on identifying undetected or undiagnosed cases among those with one or more of the four preselected conditions to initiate treatment. Additionally, the study seems to exclude individuals already hospitalized at the start of the study period. However, prevalence rates are consistently mentioned throughout the protocol, does the study intend to focus instead on estimating incidence rates?
3. Could the authors clarify the definition of "acute medical hospital admissions" and explain whether the specific health reason for hospital admission is a predictor of interest in the analysis, and if so, how will it be utilized?
4. The study's exclusive recruitment of patients admitted to emergency care for acute medical hospital admissions suggests a focus solely on severe-stage patients. This approach represents a missed opportunity to identify individuals in earlier stages of disease progression and enable their linkages to care.
5. Would a person be enrolled in the study if they were discharged on the same day as their emergency admission?
6. The criteria of including only participants who are contactable on discharge by phone inadvertently marginalizes groups lacking access to phones/technology.

**Study setting:**

1. Could the authors please provide additional information regarding the urban or rural context of the hospitals selected as study sites and provide more information on the approximate size of the catchment area served by each of the selected hospitals in Malawi and Tanzania? Additionally, how do factors such as population density and accessibility to healthcare services vary across the catchment areas of these hospitals?

**Scope of multimorbidity:**

1. The scope of multimorbidity prevalence assessment in this project is limited, focusing solely on four preselected conditions: high blood pressure, diabetes, HIV, and chronic kidney disease. However, Fortin et al. advocated for a broader approach and proposed including at least 12 conditions to assess multimorbidity prevalence (Fortin *et al.*, 2012 ^3^). By incorporating a more comprehensive range of conditions, biases and underestimations can be minimized, providing a more accurate representation of the true burden of multimorbidity within the studied population.
2. The plain summary, as well as other sections, mentioned that "The cohort study aims to determine multimorbidity prevalence". However, considering the narrow scope of the study focusing on a limited set of chronic conditions, it might be more accurate to reflect this through precise wording throughout the protocol. Therefore, instead of "multimorbidity prevalence", it could be more appropriate to use phrases like "common comorbidity occurrences in hospital-admitted patients" or "a limited scope of multimorbidity prevalence assessment based on a list of four preselected high-burden/high-prevalent chronic conditions". This adjustment ensures alignment between the study's objectives and the terminology used throughout the protocol.
3. The prevalence of mental health disorders among patients with multimorbidity ( https://moseschikoti.com/wp-content/uploads/2019/07/Multimorbidity-and-depression-A-systematic-review-and-meta-analysis.pdf), the bidirectional interplay between depression and multimorbidity (Triolo *et al.*, 2020 ^4^), and the significant impact/treatment burden of physical-mental health multimorbidity (Cicek *et al.*, 2022 ^5^) are widely recognized in multimorbidity research. Particularly in sub-Saharan Africa, mental health conditions are frequently undiagnosed and undertreated. Depression's pivotal role in multimorbidity patterns justifies focused screening and treatment for at-risk individuals (Birk *et al.*, 2019 ^6^). However, while outcomes related to mental health may be partially captured through instruments like the EQ5D and the Patient Health Questionnaire (PHQ)-9 for depression (a rapid assessment tool with various limitations, Eack *et al.*, 2006 ^7^), their importance warrants greater attention and focus. Presently, mental disorders are neither included in the list of selected conditions for the assessment of multimorbidity prevalence nor identified as a primary clinical outcome of interest in the study, underscoring the need for its inclusion to ensure a comprehensive assessment of multimorbidity prevalence, patient health status and outcomes.

**Outcomes of interest:**

1. While the below named study is currently only available in preprint format, it will soon be published in BMJ Global Health. The reviewer recommends that the authors look into this study and consider integrating core outcome sets tailored for trials of interventions aimed at preventing and treating multimorbidity in low- and middle-income countries. Notably, "adherence to treatment" appears to be absent from the current protocol and merits inclusion to comprehensively evaluate intervention efficacy.
  Vidyasagaran *et al.* (2024 ^8^).
  A comprehensive Core Outcome Set (COS) for multimorbidity, particularly tailored to low- and middle-income countries (LMICs), is lacking. This study followed stringent guidelines to develop COS specific to LMICs. The research identified four essential outcomes for prevention interventions and four for treatment interventions in LMIC settings. These outcomes encompass adverse events, quality of life, development of new comorbidities, health risk behavior, adherence to treatment, and out-of-pocket expenditure. A context-sensitive COS has the potential to minimize research redundancy, standardize outcome measures, and propel multimorbidity research in LMICs, ultimately improving health outcomes for people living with multimorbidity.

**Data collection:**

1. Will there also be an EQ5D5L questionnaire administered at point of discharge (E)? What is shown for EQ5D5L at timepoint E (discharge) in Table 1 do not align with the following texts: “The questionnaire will be administered, at admission, discharge, 30 and 90 days post admission in person or (day 30) by phone (as for patient costs).”
2. What’s the difference between structured discussions and focused group interviews (Table 2)?
3. Could the authors please provide more details regarding the measures that will be taken to ensure the objectivity and reliability of the structured observations conducted during patient admission - specifically, any steps taken to minimize potential biases in this data collection process?

**Analysis plan:**

1. What statistical methodologies will be employed to estimate the costs associated with specific combinations of morbidity, ensuring that the influence of other comorbidities and covariates is effectively controlled for?

**Other:**

1. In the context of multimorbidity, when discussing various combinations of co-existing conditions, it may be more appropriate to use terms such as "disease combinations", "morbidity combinations" or, in the case of two conditions, "pairs/dyads" and in the case of three, "triads", etc. The term "multimorbidity combination" being used lacks phrasal-semantic coherence.
2. Minor: Some abbreviations were not defined at first instance of use, but later on (IDI, FGD, HCW…) and some were not defined at all.
3. Minor: Various closing brackets missing.

Is the study design appropriate for the research question?

Partly

Is the rationale for, and objectives of, the study clearly described?

Yes

Are sufficient details of the methods provided to allow replication by others?

Partly

Are the datasets clearly presented in a useable and accessible format?

Not applicable

Reviewer Expertise:

My work focuses on health economics, primary care, infectious diseases, and multimorbidity.

I confirm that I have read this submission and believe that I have an appropriate level of expertise to confirm that it is of an acceptable scientific standard, however I have significant reservations, as outlined above.

## References

[1] : Informing 'good' global health research partnerships: A scoping review of guiding principles. *Glob Health Action* .2021;14(1) : 10.1080/16549716.2021.1892308 1892308 10.1080/16549716.2021.1892308 33704024 PMC7954413

[2] : Embracing complexity and uncertainty to create impact: exploring the processes and transformative potential of co-produced research through development of a social impact model. *Health Res Policy Syst* .2018;16(1) : 10.1186/s12961-018-0375-0 118 10.1186/s12961-018-0375-0 30537975 PMC6288891

[3] : A systematic review of prevalence studies on multimorbidity: toward a more uniform methodology. *Ann Fam Med* .2012;10(2) : 10.1370/afm.1337 142-51 10.1370/afm.1337 22412006 PMC3315131

[4] : The complex interplay between depression and multimorbidity in late life: risks and pathways. *Mech Ageing Dev* .2020;192: 10.1016/j.mad.2020.111383 111383 10.1016/j.mad.2020.111383 33045250

[5] : Depression and unplanned secondary healthcare use in patients with multimorbidity: A systematic review. *PLoS One* .2022;17(4) : 10.1371/journal.pone.0266605 e0266605 10.1371/journal.pone.0266605 35390086 PMC8989325

[6] : Depression and multimorbidity: Considering temporal characteristics of the associations between depression and multiple chronic diseases. *Health Psychol* .2019;38(9) : 10.1037/hea0000737 802-811 10.1037/hea0000737 31008648 PMC6706317

[7] : Limitations of the Patient Health Questionnaire in Identifying Anxiety and Depression: Many Cases Are Undetected. *Res Soc Work Pract* .2006;16(6) : 10.1177/1049731506291582 625-631 10.1177/1049731506291582 24465121 PMC3899353

[8] : Core outcome sets for trials of interventions to prevent and to treat multimorbidity in low- and middle-income countries: the COSMOS study. *medRxiv* .2024; 10.1101/2024.01.29.24301589 10.1101/2024.01.29.24301589 PMC1134021639160083

# Reviewer report: Reviewer response for version 1

**DOI:** 10.3310/nihropenres.14663.r31103

**Published:** 2024-04-12

**Authors:** Claire Rosen

**Affiliations:** 1 University of Pennsylvania, Philadelphia, PA, USA

The proposed study protocol outlines, in detail, the rational behind the first two phases of a three-phase mixed-methods investigation to:

1. Describe the prevalence of HIV, hypertension, diabetes, and chronic kidney disease among patients admitted to 4 hospitals (two in Malawi and two in Tanzania) over the span of one calendar year and to examine the association between these comorbidities and clinical outcomes.
2. Measure and describe the costs incurred and changes in health-related quality of life at 90 days post-admission
3. Qualitatively study patients and carer's experience within the healthcare system, and specifically regarding the impact of common comorbidities on care seeking, care navigation, and care adherence. 

I would like to begin by applauding the investigators on this well thought-out research proposal, and specifically for their robust community engagement in designing the study. I am impressed by breadth of investigation that the authors plan to undertake. 

 I have a few small suggestions that I think would improve this project. To start, the definition of multimorbidity in the literature is not strictly defined as "two or more comorbidities". In fact, there has been a decent amount of debate in the literature over the ideal definition of multimorbidity. Furthermore, the authors are specifically looking at HIV, hypertension, diabetes, and chronic kidney disease. As such, it would be more honest of an approach to describe the aims and study to be that of understanding the prevalence of specific common comorbid conditions to the populations of interest, and the impact of those comorbidities on outcomes. Either way, a clarification that "multimorbidity" is not universally defined (e.g. "though there are multiple proposed definitions of multimorbidity, we choose to define multimorbidity as X because Y") and that the study is specifically looking at diseases of importance to the study population (HIV, hypertension, diabetes, and chronic kidney disease) is necessary. 

 In your methods for this study, you mention that you will include patients with a "physician diagnosis of an acute medical disorder". How will this be judged? Is it from the clinical records? Claims data? Do you have a proposed list of medical disorders that you plan to include?

 Finally, in your plans for Phase 1a analysis, you note that you plan to use logistic regression. It would be helpful to clearly define your planned covariates, or how you intend to select covariates for your regression model. Further, you mention multiple outcomes - as such, would be sure to clarify how you will define statistical or clinical significance, and how this will be adjusted for multiple outcomes (e.g. Bonferroni). 

 Thank you for the opportunity to review your planned investigation - I wish you the best of luck with your endeavors!

Is the study design appropriate for the research question?

Yes

Is the rationale for, and objectives of, the study clearly described?

Yes

Are sufficient details of the methods provided to allow replication by others?

Yes

Are the datasets clearly presented in a useable and accessible format?

Not applicable

Reviewer Expertise:

Health services research and multimorbidity

I confirm that I have read this submission and believe that I have an appropriate level of expertise to confirm that it is of an acceptable scientific standard, however I have significant reservations, as outlined above.

# Response

**Published:** 2024-04-22

**Authors:** Stephen Spencer

**Affiliations:** Department of Clinical Science, Liverpool School of Tropical Medicine, UK

**Reviewer’s comment**

 I have a few small suggestions that I think would improve this project. To start, the definition of multimorbidity in the literature is not strictly defined as "two or more comorbidities". In fact, there has been a decent amount of debate in the literature over the ideal definition of multimorbidity. Furthermore, the authors are specifically looking at HIV, hypertension, diabetes, and chronic kidney disease. As such, it would be more honest of an approach to describe the aims and study to be that of understanding the prevalence of specific common comorbid conditions to the populations of interest, and the impact of those comorbidities on outcomes. Either way, a clarification that "multimorbidity" is not universally defined (e.g. "though there are multiple proposed definitions of multimorbidity, we choose to define multimorbidity as X because Y") and that the study is specifically looking at diseases of importance to the study population (HIV, hypertension, diabetes, and chronic kidney disease) is necessary.

**Authors’ reply**

*Thank you very much for your suggestion. We agree and have modified the final paragraph in the introduction section;  we have highlighted that there limitations with the definition, particularly to different contexts (and provided an additional reference); and clarified that we will focus on HIV, hypertension, diabetes and chronic kidney disease.*

**Reviewer’s comment**

 In your methods for this study, you mention that you will include patients with a "physician diagnosis of an acute medical disorder". How will this be judged? Is it from the clinical records? Claims data? Do you have a proposed list of medical disorders that you plan to include?

**Authors’ reply**

*Thank you for highlighting this was not clear. Inclusion was based on capture of acute medical disorder from medical records, using ICD codes. The Inclusion criteria in the manuscript has been updated to reflect this.*

**Reviewer’s comment**

 Finally, in your plans for Phase 1a analysis, you note that you plan to use logistic regression. It would be helpful to clearly define your planned covariates, or how you intend to select covariates for your regression model. Further, you mention multiple outcomes - as such, would be sure to clarify how you will define statistical or clinical significance, and how this will be adjusted for multiple outcomes (e.g. Bonferroni).

**Authors’ reply**

*Thank you for raising this. We will publish a statistical analysis plan (SAP) as a component of our reporting, which is over-seen by the study statistician (Dr Sarah White). We plan to publish this separately on an online repository (see updated ‘Analysis’ section).*

# Reviewer report: Reviewer response for version 1

**DOI:** 10.3310/nihropenres.14663.r31181

**Published:** 2024-03-14

**Authors:** Rifqah Abeeda Roomaney

**Affiliations:** 1 South African Medical Research Council, Cape Town, South Africa

Thank you for the opportunity to review the study protocol titled “Multimorbidity-associated emergency hospital admissions: a “screen and link” strategy to improve outcomes for high-risk patients in sub-Saharan Africa: a prospective multicentre cohort study protocol” by Spencer *et al.*, in consideration of publication in the NIHR Open Research Journal.

 Multi-morbidity plays an important role in the disease burden in Africa. I am unaware of such a large-scale study that has taken place in Sub-Saharan Africa and thus the authors are addressing a knowledge gap i.e. multi-morbidity in emergency hospital admissions and the assessment of HRQoL and the related economic costs.

 As far as I am aware, injuries have not been well incorporated into studies of multi-morbidity. Given that the setting is hospital-based, the authors may want to incorporate some information on injuries in their analysis.  

 The protocol is well-written, thorough and clear. I commend the authors for publishing a protocol, so that similar studies could theoretically be performed elsewhere. I look forward to reading the outcomes of the study. This study will be a great addition to the knowledge base in sub-Saharan Africa and answer many important questions.

 Minor:

 Introduction

 - Can the authors elaborate on why there has been an increase in life expectancy in Malawi and Tanzania? Although I can guess why, it would be better for the authors to state.

 Objectives

 - The authors have justified why they are interested in certain pre-selected diseases. They may want to consider the existence of other diseases (even if self-report) to control for confounding in the analysis. The same goes for body mass index. I note that this is addressed by the authors in the limitations.  

 -  Can the authors elaborate on why admissions for primary trauma are excluded?

Is the study design appropriate for the research question?

Yes

Is the rationale for, and objectives of, the study clearly described?

Yes

Are sufficient details of the methods provided to allow replication by others?

Yes

Are the datasets clearly presented in a useable and accessible format?

Not applicable

Reviewer Expertise:

Multimorbidity, injuries and risk factors

I confirm that I have read this submission and believe that I have an appropriate level of expertise to confirm that it is of an acceptable scientific standard.

# Response

**Published:** 2024-04-22

**Authors:** Stephen Spencer

**Affiliations:** Department of Clinical Science, Liverpool School of Tropical Medicine, UK

*We would like to take the opportunity to thank the reviewer for their time and thoughtful consideration. We have carefully addressed all comments and updated the manuscript which we believe is now much improved.*

**Reviewer’s comment**

 Introduction

 Can the authors elaborate on why there has been an increase in life expectancy in Malawi and Tanzania? Although I can guess why, it would be better for the authors to state.

**Authors’ reply**

*Thank you for this suggestion. We have provided explanations (with references) for improvements in life expectancy in Malawi and Tanzania (improvements in prevention and treatment for infectious diseases, particularly HIV/AIDS, Tuberculosis and Malaria, as well as improvements in maternal and child survival).*

**Reviewer’s comment**

 Objectives

 The authors have justified why they are interested in certain pre-selected diseases. They may want to consider the existence of other diseases (even if self-report) to control for confounding in the analysis. The same goes for body mass index. I note that this is addressed by the authors in the limitations. 

**Authors’ reply**

*We agree. In the objectives section we have highlighted that we are focussing on the four pre-selected diseases using gold-standard diagnostic criteria to inform the design of our planned trial. We have added an additional objective: to measure the prevalence of self-reported / clinically-reported chronic diseases.*

**Reviewer’s comment**

 Can the authors elaborate on why admissions for primary trauma are excluded?

**Authors’ reply**

*We acknowledge and agree that trauma is an increasing burden of health systems and an important area to develop context-sensitive policies to improve outcomes. We opted to focus on medical populations as there are existing studies in this context (e.g.* *https://doi.org/10.1016/S2214-109X(23)00346-7* *; doi: 10.2471/BLT.23.290755), and health system interventions are for these groups are being addressed. We have made this clearer in the list of exclusions.*
